# ACTIVITY LEVEL AND SUCCESSFUL AGING, SUBJECTIVE AGE, AND MENTAL, PHYSICAL, AND GENERAL HEALTH

**DOI:** 10.1093/geroni/igad104.2304

**Published:** 2023-12-21

**Authors:** Isabel Mancilla, Lucia Rivera, Jaclyn Bergstrom, Anthony Molina

**Affiliations:** University of California San Diego, San Diego, California, United States; University of California, San Diego, San Diego, California, United States; University of California, San Diego, San Diego, California, United States; University of California, San Diego, San Diego, California, United States

## Abstract

Perceived well-being refers to an individual’s subjective evaluation of their quality of life. Importantly, perceived well-being takes into account an individual’s emotional, social, and psychological health, which are all critical components of a fulfilling and satisfying life. The purpose of this study is to evaluate the correlations between activity level from the International Physical Activity Questionnaire (IPAQ) and self-reported outcomes related to perceived well-being (successful aging, subjective age discrepancy), and mental, physical, and general health outcomes from the 36 Item Short Form Survey (SF-36). We utilized existing data from 595 individuals aged 50 years or older enrolled in UC San Diego’s Successful Aging Evaluation (SAGE) study. Participants were stratified into low, medium, and high activity levels from the IPAQ. Groups were then compared to self-rated successful aging, subjective age questions, and the SF-36. Our results indicate that increased physical activity is positively associated with subjective successful aging. We observed a significant linear trend in IPAQ physical activity correlated to mental health (p=.0242), physical health (p=<.0001), and general health (p=(<.0001). A possible limitation of this study includes that of the 595 participants, 81% of participants identified as Caucasian. It is critical for future studies to incorporate a more diverse sample size to accurately present future findings. Understanding the factors that influence perceived well-being can lead to strategies for improving the overall outlook to support successful aging.

